# T15. LONGITUDINAL ASSOCIATIONS BETWEEN CHILDHOOD SALIVARY CORTISOL LEVELS AND PRODROMAL SYMPTOMS IN LATE ADOLESCENCE: FINDINGS FROM A HIGH-RISK COHORT

**DOI:** 10.1093/schbul/sby016.291

**Published:** 2018-04-01

**Authors:** Alexis Cullen, Elizabeth Fraser, Helen Fisher, Ruth Roberts, Uzma Zahid, Carmine Pariante, Patricia Zunszain, Philip McGuire, Robin Murray, Valeria Mondelli, Kristin R Laurens

**Affiliations:** 1 Institute of Psychiatry, Psychology & Neuroscience, King’s College London; 2 Australian Catholic University

## Abstract

**Background:**

Individuals with established psychosis are characterised by a distinct pattern of hypothalamic-pituitary-adrenal (HPA) axis dysfunctions which include both elevated daytime cortisol levels and a blunted cortisol awakening response (CAR). Whilst these patterns of dysfunction have also been observed among those at elevated risk for the disorder, longitudinal studies are scarce. As such, the relevance of these HPA axis abnormalities for the progression of psychopathology in high-risk populations is unknown. Utilising data from a well-characterised, longitudinal cohort of youth at elevated risk for schizophrenia and their typically-developing peers (The Child Health and Development Study), we aimed to investigate the extent to which HPA axis function determined in childhood is a significant predictor of putative prodromal status and psychopathology in late adolescence/early adulthood.

**Methods:**

The sample comprised high-risk individuals who presented either multiple antecedents of schizophrenia (developmental delays, psychopathology, and psychotic-like experiences: ASz=21) or a family history of illness (FHx=13), and typically-developing youth with neither antecedents nor a family history (TD=36). Participants were recruited at age 9–12 years using a community screening method and assessed biennially throughout adolescence. At the age 11–14 years assessment phase, participants collected salivary cortisol samples in their home environment which were used to determine diurnal cortisol secretion and the CAR. At the age 17–21 years assessment phase, participants completed measures of prodromal symptoms (Prodromal Questionnaire: PQ), depression (Quick Inventory of Depressive Symptomatology questionnaire: QIDS), and anxiety (Social Interaction Anxiety Scale: SIAS). Established PQ thresholds were used to identify participants who met probable prodromal status. Logistic and linear regression analyses were used to examine the extent to which salivary cortisol measures at age 11–14 years predicted probable prodromal status and continuous psychopathology measures at 17–21 years, respectively.

**Results:**

Relative to the TD group, ASz youth were characterised by higher depression (B=0.24, p=0.05) and disorganised symptoms (B=0.36, p=0.007) at 17–21 years whilst FHx youth obtained higher scores on the PQ general symptoms scale (B=0.24, p=0.048). Analyses performed in the total sample indicated that the CAR was negatively associated with depression symptoms (B=-0.28, p=0.006) and PQ negative symptoms (B=-0.30, p=0.004) at age 17–21 years. Positive associations were observed between diurnal cortisol and positive (B=0.41, p=0.02), disorganised (B=0.30, p=0.04), and general (B=0.29, p=0.03) PQ symptoms. Diurnal cortisol levels were also significantly associated with probable prodromal status at follow-up (OR=1.04, p=0.04). No significant interactions were observed between group status and salivary cortisol levels in any model. After adjustment for potential confounders (age, follow-up time, sex, BMI, and pubertal status), the CAR continued to show significant associations with both depression (p=0.006) and PQ negative symptoms (p=0.007) whilst only a statistical trend was observed for the relationship between diurnal cortisol levels and positive symptoms (p=0.055).

**Discussion:**

The current study is the first to examine the extent to which HPA axis function can predict development of prodromal symptoms in a high-risk cohort. Our finding that more abnormal HPA axis function (i.e., a decreased CAR and higher diurnal cortisol) at age 11–14 years is associated with both prodromal and depression symptoms at age 17–21 has important implications for aetiological theories and for clinical practice.

